# Fournier's Gangrene of the Penis: A Rare Entity

**DOI:** 10.4103/0974-2077.63394

**Published:** 2010

**Authors:** Ashutosh Talwar, Neerja Puri, Majhail Singh

**Affiliations:** *Department of Surgery and Dermatology, GGS Medical College and Hospital, Faridkot-151 203, Punjab, India*

**Keywords:** Anaerobes, antibiotics, debridement, Fournier's gangrene, penis, scrotum

## Abstract

Fournier's gangrene is a rare, fulminant, but and usually a localized disease of the scrotum and penis, with an occasional extension up to the abdominal wall. The usual organism is an anaerobic streptococcus synergistic with other organisms. A 45-year-old male presented with fever and pain and a brownish-black discolouration of the penis, of four days. Our case was unusual in that the penis was involved, which is very rare. Early therapy is the key, including hospitalization, debridement of the entire shaft of the penis distal to the devastated area, without excising the normal skin, parenteral broad-spectrum antibiotics, and skin grafting.

## INTRODUCTION

Fournier's gangrene is a necrotizing infection that involves the soft tissues of the male genitalia.[1,2] Fournier's gangrene is a specific form of necrotizing fasciitis, a general term introduced in 1951, by Wilson, to describe infection of the soft tissue, which involves the deep and superficial fascia, regardless of the location. Originally, the term Fournier's gangrene was used to describe idiopathic gangrene of the genitalia; however, it has also been used to describe most soft tissue necrotizing infections of the perineum, independent of the cause. Modern day use of the term Fournier's gangrene should be restricted to describe infections that primarily involve the genitalia.

In his presentation, Fournier reviews the systemic and local factors that influence this fulminative process. Local factors related to the trauma of the genitalia accounted for a vast majority of the cases of genital gangrene. Although Fournier has not emphasized the role of diabetes in this article, diabetes was known as the leading predisposing systemic factor.[3] Fournier describes in anecdotes some of the misconceptions of the times that created this condition, including the practice of nighttime ligation of the prepuce to control enuresis or an attempted birth control technique practiced by an adulterating man to avoid impregnating his married lover. Since Fournier's description, subsequent knowledge has shown that it has an identifiable cause, which frequently manifests in a more indolent fashion. Trauma to the genitalia continues to be a frequently recognized vector for the introduction of bacteria that initiate the infectious process.[4]

## CASE REPORT

**Figure 1:**
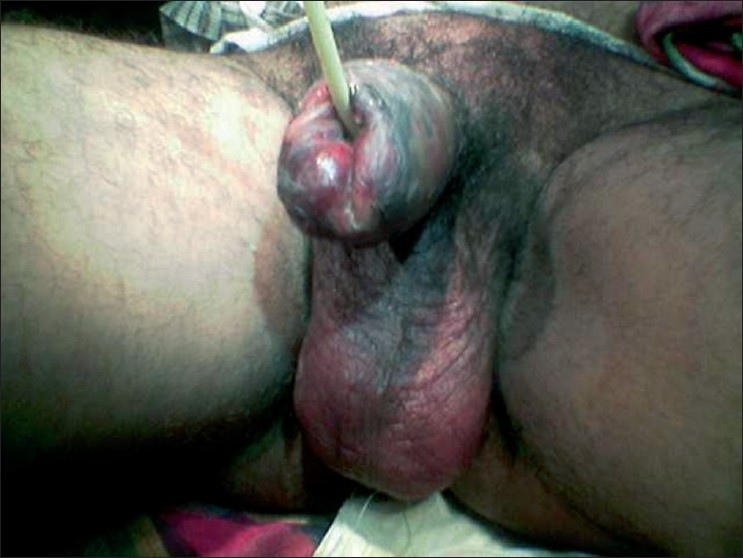
Brownish-black discolouration of the penis with erythema of the scrotum

**Figure 2:**
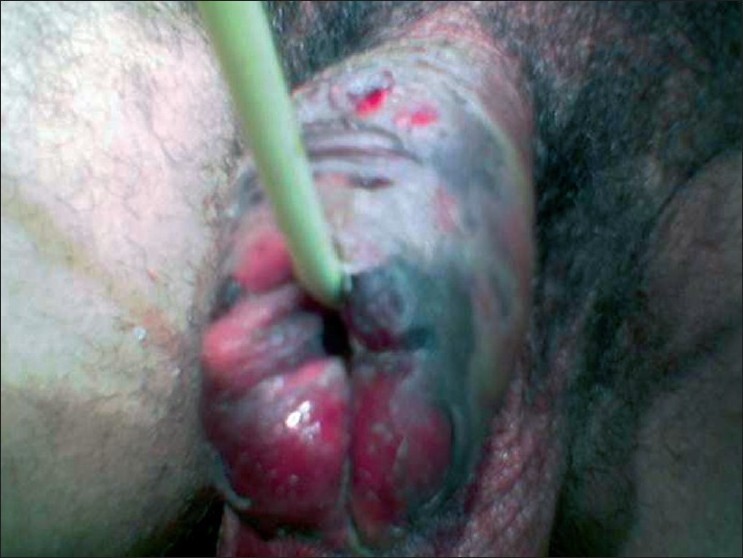
Discolouration of the penis with vesicles filled with hemorrhagic fluid

A 45 year-old-male patient presented with fever and pain, with brownish black discolouration of the penis [Figure 1] for four days. There was no history of trauma or any sepsis in the genitoperineal area. On general physical examination, the patient was febrile. Local examination revealed brownish-black discoloration of the penile skin extending up to the penoscrotal junction without any clear line of demarcation [Figure 2]. Multiple vesicles filled with hemorrhagic fluid were present over the penis. There was associated erythema of the scrotum. There were no other foci of infection in the genitoperineal area. The prostate gland was normal on rectal examination. Routine hematological examination revealed leucocytosis and neutrophilia. Urine microscopy revealed no abnormality. Random blood sugar, blood urea, and serum creatinine were within normal limits. The ultrasonological examination of the abdomen and pelvis was normal. Discharge sent for culture isolated a mixed growth of *Bacteroides fragilis*, *Bacteroides fragilis* and anaerobic *streptococcus* sensitive to cefotaxime, ceftriaxone, amoxicillin/ clauvulonic acid and amikacin.

**Figure 3:**
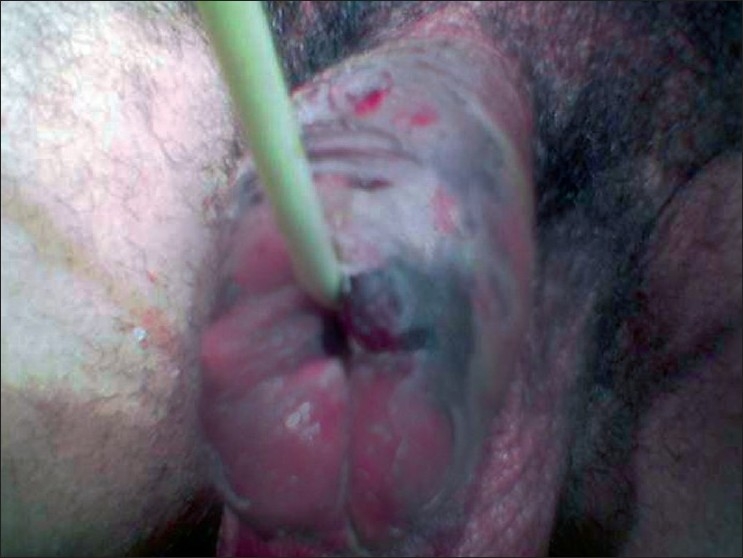
Penile skin after debridement

The patient was hospitalized and broad spectrum antibiotics including cefotaxime and metronidazole were administered parenterally. Emergency multiple decompressing incisions were placed over the gangrenous penile skin and the inflammatory fluid was drained. Two days later, debridement was done. After repeated debridement and dressings the bed was finally healthy [Figure 3], and an unexpanded, meshed, split-thickness skin graft was performed, by placing the graft junction on the ventral surface of the penis.[5] The graft dressing was changed on the fourth and sixth postoperative days, and it revealed a 100% take of the graft. The postoperative period was uneventful.

## DISCUSSION

Fournier's gangrene is a rare, fulminant, and usually localized disease of the scrotum and penis, with occasional extension up to the abdominal wall. The usual organism is an anaerobic streptococcus synergistic with other organisms like *enterobacteria* species, *staphylococcal* species, anerobic organisms and fungi. Our case was unusual in that only the penis was involved, without any involvement of the scrotum or abdominal wall. Early therapy is the key, including hospitalization, debridement of the entire shaft of the penis distal to the devastated area without excising the normal skin, parenteral broad-spectrum antibiotics and skin grafting. Only few cases of Fournier's gangrene of the penis have been reported.[6–9] The following are the pathognomonic findings on the pathological evaluation of the involved tissue:

- Necrosis of the superficial and deep fascial planes
- Fibrinoid coagulation of the nutrient arterioles
  - Polymorphonuclear cell infiltration
  - Microorganisms identified within the involved tissues
  - Air in the perineal tissues

Infection represents an imbalance in host immunity, which is frequently compromised by one or more of the above-mentioned co-morbid systemic processes and the virulence of the causative microorganisms. The aetiological factors allow the portal for entry of the microorganism into the perineum. The compromised immunity provides a favourable environment to initiate the infection and the virulence of the microorganism promotes the rapid spread of the disease.[8] Microorganism virulence results from the production of toxins or enzymes that create an environment conducive to rapid microbial multiplication. In a 1924 series of Chinese men with necrotizing infections, Meleney reported that the predominant organisms recovered from the cultures were the streptococcal species. Meleney attributed the necrotizing infection to this sole organism; however, subsequent clinical series have emphasized the multi-organism nature of most cases of necrotizing infection, including Fournier's gangrene. At present, recovering only *streptococcal* species is unusual; rather, *streptococcal* organisms are cultured along with as many as five other organisms. The following are the common causative microorganisms: *streptococcal* species, *staphylococcal* species, *enterobacteriaceae* species, anaerobic organisms and fungi. Most authorities believe the polymicrobial nature of this disease is necessary to create the synergy of enzyme production that promotes rapid multiplication and spread of the infection. For example, one microorganism might produce the enzymes necessary to cause coagulation of the nutrient vessels. Thrombosis of these nutrient vessels reduces local blood supply; thus, the tissue oxygen tension falls. The resultant tissue hypoxia allows growth of facultative anaerobes and microaerophilic organisms. The latter microorganisms, in turn, may produce enzymes (e.g., lecithinase, collagenase), which lead to the digestion of fascial barriers, thus fueling the rapid extension of the infection. Fascial necrosis and digestion are the hallmarks of this disease process; this is important to appreciate because it provides the surgeon with a clinical marker of the extent of tissue involvement. Specifically, if the fascial plane can be separated easily from the surrounding tissue by blunt dissection, it is quite likely to be involved with the ischemic-infectious process; therefore, any such dissected tissue should be excised. Far-advanced or fulminant disease can spread from the fascial envelopment of the genitalia throughout the perineum, along the torso, and occasionally, into the thighs.

The hallmark of Fournier's gangrene is intense pain and tenderness in the genitalia.[8] The clinical course usually progresses through the following phases:

- Prodromal symptoms of fever and lethargy, which may be present for two to seven days
- Intense genital pain and tenderness that is usually associated with edema of the overlying skin
- Increasing genital pain and tenderness with progressive erythema of the overlying skin
- Dusky appearance of the overlying skin; subcutaneous crepitation
- Obvious gangrene of a portion of the genitalia; purulent drainage from the wounds

The systemic effects of this process vary from local tenderness with no toxicity to florid septic shock. In general, the greater the degree of necrosis the more profound the systemic effects. The typical patient would be an elderly male in his sixth or seventh decade of life with comorbid diseases; females are not immune to this disease, but are affected less frequently. The characteristic histological finding that most commonly indicates Fournier's disease is fibrinoid thrombosis of the nutrient vessels that supply the superficial and deep fascia. A frequent occurrence is a widespread necrosis of the fascia with acute inflammatory cell infiltration, necrotic debris and frequent demonstration of causative microorganisms within the tissues. This extensive inflammatory process is often present deep in the intact skin, which is often minimally involved with the inflammatory process until late in the disease. Treatment also involves the institution of broad-spectrum antibiotic therapy. The antibiotic spectrum should cover *staphylococci, streptococci,* the *Enterobacteriaceae* family of organisms and anaerobes. A reasonable empiric regimen might consist of ciprofloxacin and clindamycin. Clindamycin is particularly useful in the treatment of necrotizing soft tissue infections, due to its gram-positive and anaerobic spectrum of activity. Clindamycin has been shown, in animal models of streptococcal infection, to have superior response rates compared to penicillin or erythromycin, even though the treatment is delayed.[10,11]If the initial tissue stains show fungi, an empiric antifungal agent such as amphotericin B is added. In cases associated with the sepsis syndrome, therapy with intravenous immunoglobulin (IVIG), which is thought to neutralize superantigens such as the streptotoxins (A, B) believed to mitigate the exaggerated cytokine response, has been shown to be a good adjuvant to appropriate antibiotic coverage and complete surgical debridement.[12]

Only few cases of Fournier's gangrene of the penis have been reported so far. This case is reported because of its rarity.
